# HDL Improves Cholesterol and Glucose Homeostasis and Reduces Atherosclerosis in Diabetes-Associated Atherosclerosis

**DOI:** 10.1155/2021/6668506

**Published:** 2021-05-06

**Authors:** Belinda A. Di Bartolo, Siân P. Cartland, Scott Genner, Pradeep Manuneedhi Cholan, Melissa Vellozzi, Kerry-Anne Rye, Mary M. Kavurma

**Affiliations:** ^1^The University of Sydney, Kolling Institute of Medical Research, Sydney, Australia; ^2^Faculty of Medicine and Health, Sydney, Australia; ^3^Heart Research Institute, Sydney, Australia; ^4^The University of New South Wales, Faculty of Medicine, Sydney, Australia

## Abstract

**Background and Aims:**

Apolipoprotein A-I (ApoA-I), the main component of high-density lipoprotein (HDL), not only promotes reverse cholesterol transport (RCT) in atherosclerosis but also increases insulin secretion in pancreatic *β*-cells, suggesting that interventions which raise HDL levels may be beneficial in diabetes-associated cardiovascular disease (CVD). Previously, we showed that TNF-related apoptosis-inducing ligand (TRAIL) deletion in *Apolipoprotein E*knockout (*Apoe^−/−^*) mice results in diabetes-accelerated atherosclerosis in response to a “Western” diet. Here, we sought to identify whether reconstituted HDL (rHDL) could improve features of diabetes-associated CVD in *Trail^−/−^Apoe^−/−^* mice.

**Methods and Results:**

*Trail^−/−^Apoe^−/−^* and *Apoe^−/−^* mice on a “Western” diet for 12 weeks received 3 weekly infusions of either PBS (vehicle) or rHDL (containing ApoA-I (20 mg/kg) and 1-palmitoyl-2-linoleoyl phosphatidylcholine). Administration of rHDL reduced total plasma cholesterol, triglyceride, and glucose levels in *Trail^−/−^Apoe^−/−^* but not in *Apoe^−/−^* mice, with no change in weight gain observed. rHDL treatment also improved glucose clearance in response to insulin and glucose tolerance tests. Immunohistological analysis of pancreata revealed increased insulin expression/production and a reduction in macrophage infiltration in mice with TRAIL deletion. Furthermore, atherosclerotic plaque size in *Trail^−/−^Apoe^−/−^* mice was significantly reduced associating with increased expression of the M2 macrophage marker CD206, suggesting HDL's involvement in the polarization of macrophages. rHDL also increased vascular mRNA expression of RCT transporters, ABCA1 and ABCG1, in *Trail^−/−^Apoe^−/−^* but not in *Apoe^−/−^* mice*. Conclusions*. rHDL improves features of diabetes-associated atherosclerosis in mice. These findings support the therapeutic potential of rHDL in the treatment of atherosclerosis and associated diabetic complications. More studies are warranted to understand rHDL's mechanism of action.

## 1. Introduction

Diabetes is associated with an increased risk of cardiovascular diseases (CVD), including atherosclerosis. In diabetes, the pathophysiological mechanisms that promote atherosclerosis and CVD are numerous and in part still not completely clarified [1–3]. Diabetic patients have elevated plasma levels of low-density lipoprotein (LDL) and triglycerides and low levels of high-density lipoprotein (HDL) [4]. Early animal and human studies have shown HDL infusions to have favorable cardiovascular effects [5, 6], even in diabetes. Infusing reconstituted HDL (rHDL) or increasing HDL levels with overexpression of apolipoprotein A-I (ApoA-I), the major HDL apolipoprotein, reduced plaque size by altering the histological composition of experimental lesions in animal models of atherosclerosis and restenosis [7–12]. The earliest proof-of-concept studies in humans demonstrated that infusing ApoA-I, rHDL, and its delipidated form could inhibit LDL oxidation, modulate inflammation, and elicit plaque regression [13–15]. Furthermore, ApoA-I protected against diabetes by reducing stress-induced pancreatic *β*-cell apoptosis and increasing insulin secretion [16], and HDL attenuated islet cell inflammation in type 2 diabetes via ATP-binding cassette transporter-A1 and ATP-binding cassette transporter-G1 (ABCA1 and ABCG1) [16, 17]. In addition, ApoA-I improved insulin sensitivity with decreased systemic and hepatic inflammation in mice fed a high-fat diet [18]. Collectively, these suggest that interventions which raise HDL levels may be beneficial in lowering cholesterol, reducing atherosclerosis, and improving glucose homeostasis in diabetes.

TRAIL is a member of the TNF superfamily and was initially thought to selectively induce apoptosis in cancer cells. However, more recently, it has been discovered that TRAIL is implicated in coronary artery disease, acute coronary syndromes, and diabetes [19–22]. Studies have shown that TRAIL deficiency is associated with type-1 autoimmune diabetes in mice [23, 24]. Injections of soluble TRAIL receptor (an antagonist of TRAIL signaling) into nonobese diabetic (NOD) mice or streptozotocin-treated *Trail^−/−^* mice increased incidence of diabetes [23, 24]. In addition, TRAIL deficiency in mice on a “Western” style high-fat diet for 12 w markedly accelerated atherosclerosis and promoted features of diet-induced diabetes including weight gain, hyperglycemia, hypoinsulinemia, and pancreatic *β*-cell dysfunction [25]. Using this mouse model of physiologically induced diabetes-associated atherosclerosis, we sought to determine the effects of reconstituted HDL (rHDL) on improving atherosclerosis and features of diabetes.

## 2. Methods

### 2.1. rHDL Generation

HDL was isolated from autologously donated, pooled blood samples from normal healthy donors (Gribbles Pathology, South Australia, Australia) by sequential ultracentrifugation in the 1.063 < d < 1.21 g/mL density range and delipidated using standard techniques [26]. ApoA-I was isolated by anion chromatography on a Q Sepharose Fast Flow column (GE Healthcare Biosciences, Waukesha, WI, USA) attached to a fast protein liquid chromatography system [27]. Discoidal rHDLs containing ApoA-I complexed to 1-palmitoyl-2-linoleoyl phosphatidylcholine (PLPC) (Avanti Polar Lipids, Alabaster, AL, USA) were prepared using the cholate dialysis method [28]. The phospholipid : ApoA-I molar ratio was 100 : 1. The resulting rHDL was dialysed extensively against endotoxin-free phosphate-buffered saline (PBS; pH 7.4) before use.

### 2.2. Animals

Male *Trail^−/−^Apoe^−/−^* or *Apoe^−/−^* mice aged 6 weeks and weighing approximately 18 to 20 g were placed on a high-fat high-cholesterol “Western” diet (Semi-Pure Rodent Diet SF00-219; 22% fat, 0.15% cholesterol; Specialty Feeds, Glen Forrest, WA, Australia) as previously described [25]. After 10 weeks on a Western diet, mice were randomly assigned to receive either PBS vehicle or rHDL (20 mg/kg) for 2 weeks (3 times weekly). Mice were monitored daily, and body weights were recorded weekly. Mice were euthanised by cardiac exsanguination and indicated tissues isolated. All animal work was conducted according to the Animal Care and Ethics Committee guidelines, the University of New South Wales, or the Sydney Local Health District Animal Welfare Committee (Sydney, NSW, Australia).

### 2.3. Plasma and Liver Analysis

Blood was collected by cardiac puncture at the time of euthanasia. Plasma samples were stored at −80°C in EDTA-Na_2_ until required for analysis. Triacylglycerol and total cholesterol from plasma were measured using commercial kits (Wako Chemicals, Osaka, Japan). Fasting blood glucose was measured by a glucometer (Accu-check Performa; Roche, Mannheim, Germany). Insulin was measured using commercially available ELISA (Mercodia).

### 2.4. Insulin and Glucose Tolerance Tests

At 10 and 12 weeks of high-fat feeding, either 1 g/kg body weight D-glucose (Sigma-Aldrich, Sydney, NSW, Australia) was injected into mice intraperitoneally following overnight fasting or 1 U/kg body weight human insulin was injected into non-fasted mice intraperitoneally. Blood was collected by pinprick from the tail vein and plasma glucose measured using a glucometer.

### 2.5. RNA Extraction and Real-Time Quantitative PCR

Liver and thoracic aortae were snap-frozen in liquid nitrogen and stored at −80°C. Tissue was homogenised (MP Biomedicals, Sydney, NSW, Australia) and total RNA extracted in TRI reagent (Sigma) [29]. RNA was then reverse transcribed to cDNA using iSCRIPT (Bio-Rad, Sydney, NSW Australia). Real-time PCR was performed in triplicate using iQSybr Green Master Mix (Bio-Rad, Sydney, NSW Australia) in the CFX96 thermocycler (Bio-Rad, Sydney, NSW Australia). Relative changes in mRNA levels between groups were determined using the 2^−ΔΔ*C*_*t*_^ method [30]. Expression was normalised to *β*-actin, and changes were compared with *Apoe^−/−^* mice. Primer details can be found in Table 1.

### 2.6. Histology and Immunohistochemistry

Brachiocephalic arteries were processed as described [31]. Pancreata were removed and fixed in 10% formalin (wt/vol). Haematoxylin and eosin stain was used to assess tissue architecture; pancreata were stained for insulin (1 : 500; Cell Signalling) and macrophages (MAC3, 1 : 100; BD Pharmingen). All IgG controls were negative. Digital images of sections were acquired using an Olympus DP72 microscope (Olympus, Mount Waverley, Victoria, Australia) and a Zeiss Axio Imager Z2 microscope.

### 2.7. Morphometric Analysis

Morphometric analysis of plaque area : total artery area, media area :  total artery area, and necrotic core area : plaque area was performed on haematoxylin and eosin-stained sections using ImageJ (NIH, Bethesda). The percentage of positive staining in the plaque or islets was determined using cellSens Software (Olympus). Thresholds for positive staining for each antibody were determined, and sections were analysed by an investigator blinded to the mouse genotype.

### 2.8. Statistics

All results are expressed as the mean ± SEM. Statistical comparisons were made by Mann–Whitney *t-*tests and one- or two-way ANOVA, with Bonferroni's correction where appropriate. The statistics program in GraphPad Prism Version 6.0 was used (GraphPad Software, San Diego, CA, USA). A value of *p* < 0.05 was considered significant.

## 3. Results

### 3.1. HDL Reduces Plasma Glucose Levels and Improves Glucose Tolerance in Western Diet-Fed *Trail^−/−^Apoe^−/−^* Mice

**Figure 1:**
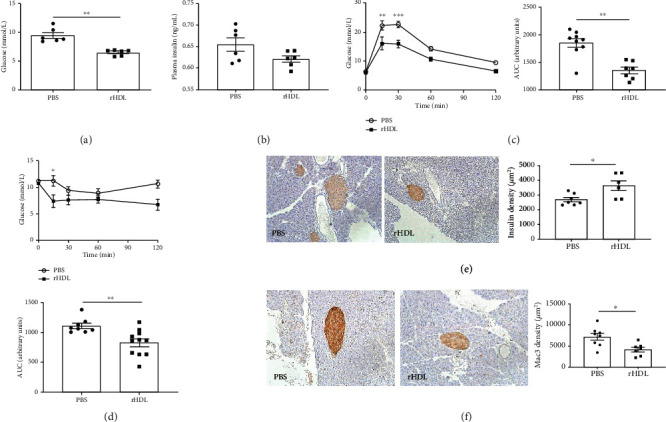
rHDL reduces plasma glucose levels and improves glucose tolerance in Western diet-fed *Trail^−/−^Apoe^−/−^* mice. *Trail^−/−^Apoe^−/−^* and *Apoe^−/−^* mice were fed a high-fat Western diet for 12 weeks. In the last 2 weeks of the study, mice received 3 weekly infusions of either rHDL (20 mg/kg) or PBS. At the conclusion of the study (a) fasting plasma glucose and (b) fasting plasma insulin were measured; *n* = 6/group. (c) Glucose and (d) insulin tolerance tests were performed as described in the methods at 12 weeks where plasma glucose levels were measured over 2 hours (*n* = 8-10). PBS-treated *Trail^−/−^Apoe^−/−^* (open circles); rHDL-treated *Trail^−/−^Apoe^−/−^* (closed squares). The area under the curve (AUC) was quantified for each. Mouse pancreatic islets were stained for (e) insulin and (f) macrophages (MAC3). Left panel: representative images of stained pancreata. Right panel: quantification (*n* = 6-8). Results are expressed as mean ± SEM; ^∗^*p* < 0.05, ^∗∗^*p* < 0.01, and ^∗∗∗^*p* < 0.001, Mann–Whitney *t-*test and ANOVA used in all conditions. rHDL: reconstituted high-density lipoprotein.

In response to a Western diet, *Trail^−/−^Apoe^−/−^* mice display features of diabetes typical of human disease [25], in particular, a marked degree of glucose intolerance. As expected, fasting glucose levels in *Trail^−/−^Apoe^−/−^* were significantly increased compared to *Apoe^−/−^* mice, associating with impaired glucose tolerance (Supplementary Figure 1A-B). Importantly, treatment with rHDL significantly reduced plasma glucose levels by ~30%, but not plasma insulin in *Trail^−/−^Apoe^−/−^* mice (Figures 1(a) and 1(b)). No changes to plasma glucose or insulin in response to rHDL in *Apoe^−/−^* mice were observed (data not shown). We next performed glucose and insulin tolerance tests (GTT and ITT) in *Trail^−/−^Apoe^−/−^* mice to assess glucose and insulin sensitivity. Vehicle-treated *Trail^−/−^Apoe^−/−^* mice had significantly increased plasma glucose levels at 15 and 30 min following a glucose challenge (Figure 1(c)), which was cleared over the 2 h timepoint. In contrast, *Trail^−/−^Apoe^−/−^* mice treated with rHDL demonstrated faster glucose clearance (Figure 1(c)). When the area under the curve was quantified, rHDL improved glucose tolerance in *Trail*^‐/‐^*Apoe*^‐/‐^ > 2-fold compared with vehicle-treated mice (Figure 1(c)). rHDL-treated mice also showed improved insulin sensitivity with greater reductions in plasma glucose at 15 min following an insulin bolus and a ~25% reduction overall when the area under the curve was quantified (Figure 1(d)). These findings were associated with a significant increase in endogenous insulin protein expression (Figure 1(e)) and reduced MAC3 staining indicating reduced macrophage content in pancreatic islets of *Trail^−/−^Apoe^−/−^* mice treated with rHDL (Figure 1(f)). In contrast, rHDL had no effect on glucose or insulin tolerance in *Apoe^−/−^* mice (Supplementary Figure 2A-B). Together, these findings suggest that rHDL improves features of Western diet-induced diabetes in *Trail^−/−^Apoe^−/−^* mice.

### 3.2. HDL Reduces Plasma Cholesterol, Triacylglycerol, and Atherosclerotic Plaque in *Trail^−/−^Apoe^−/−^* Mice

**Figure 2:**
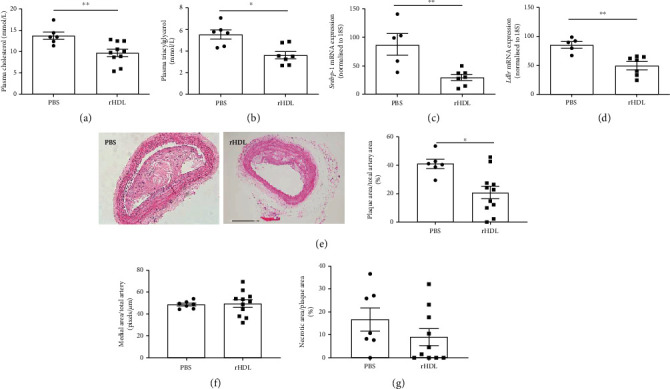
HDL reduces plasma cholesterol levels and atherosclerotic plaque in Western diet-fed *Trail^−/−^Apoe^−/−^ mice. Trail^−/−^Apoe^−/−^* and *Apoe^−/−^* mice were fed a high-fat Western diet for 12 weeks. In the last 2 weeks of the study, mice received 3 weekly infusions of either rHDL (20 mg/kg) or PBS. (a) Fasting plasma cholesterol (*n* = 6-10) and (b) triacylglycerol levels (*n* = 6) at euthanasia. rHDL reduced hepatic mRNA expression for (c) *Srebp1* and (d) *Ldlr* in 12 w *Trail^−/−^Apoe^−/−^* mice. mRNA was extracted from liver as described in Methods. mRNA expression was normalised to 18S; *n* = 5-7. (e) Right panel: representative cross-section of brachiocephalic arteries stained with haematoxylin and eosin (10x magnification). Left panel: quantification of plaque area. No change in (f) medial or (g) necrotic areas was observed (*n* = 7-11). Results are expressed as mean ± SEM; ^∗^*p* < 0.05 and ^∗∗^*p* < 0.01, Mann–Whitney *t-*test used in all conditions. rHDL: reconstituted high-density lipoprotein.

We next examined whether rHDL could improve features of CVD. Two weeks of rHDL infusion in Western diet-fed *Trail^−/−^Apoe^−/−^* mice significantly reduced plasma cholesterol (PBS 13.75 mmol/L ± 0.86 vs. rHDL 9.71 mmol/L ± 0.82; *p* < 0.01) and triacylglycerol (PBS 5.53 mmol/L ± 0.42 vs. rHDL 3.61 mmol/L ± 0.34; *p* < 0.05) levels, when compared to vehicle-treated mice (Figures 2(a) and 2(b)). We assessed mRNA expression of key genes responsible for cholesterol and lipid production, namely, sterol regulatory element-binding protein 1 (SREBP1) and low-density lipoprotein receptor (LDLR). Consistent with our findings at the plasma level, *Trail^−/−^Apoe^−/−^* mice treated with rHDL had significantly reduced mRNA expression for hepatic *Srebp1* (Figure 2(c)) and *Ldlr* (Figure 2(d)). Furthermore, rHDL infusion significantly reduced the *Trail^−/−^Apoe^−/−^* plaque area (PBS 41.02% ± 3.16 vs. rHDL 20.78% ± 4.47; *p* < 0.05Figure 2(e)), whereas no change in medial expansion or necrotic core size was evident (Figures 2(f) and 2(g)). On the other hand, rHDL had no effect on plasma cholesterol, triglycerides, or atherosclerosis plaque size in Western diet-fed *Apoe^−/−^* mice (Supplementary Figure 3A-C). These findings suggest that rHDL infusion improves features of diabetes-accelerated atherosclerosis, but not atherosclerosis alone.

### 3.3. rHDL Alters Expression of Genes Modulating Cholesterol Metabolism and Inflammation in the Vessel Wall

**Figure 3:**
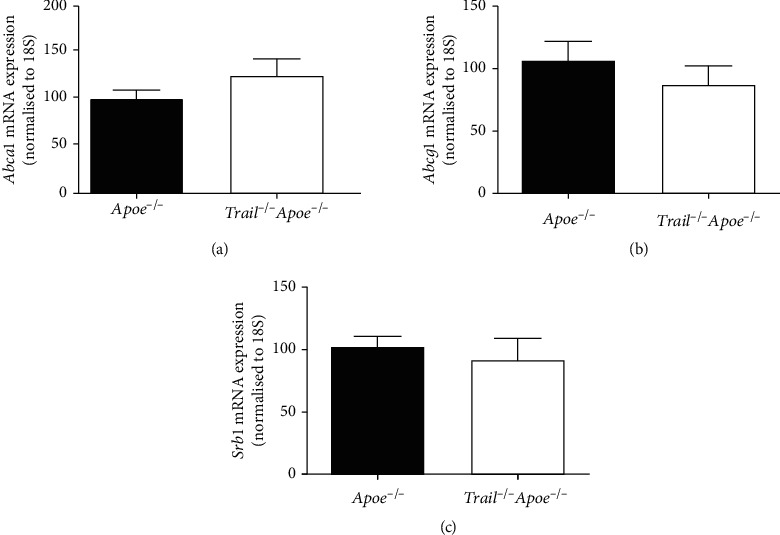
Expression of genes regulating cholesterol metabolism. *Trail^−/−^Apoe^−/−^* and *Apoe^−/−^* mice were fed a high-cholesterol diet for 12 weeks. The liver was assessed for (a) *Abca1*, (b) *Abcg1*, and (c) *Srb1* mRNA expression. mRNA was extracted from the liver as described in Methods. mRNA expression of candidate genes was normalised to 18S (*n* = 6-7). Results are expressed as the mean ± SEM; Mann–Whitney *t-*test used in all conditions. Apoe: apolipoprotein E; TRAIL: TNF-related apoptosis-inducing ligand.

**Figure 4:**
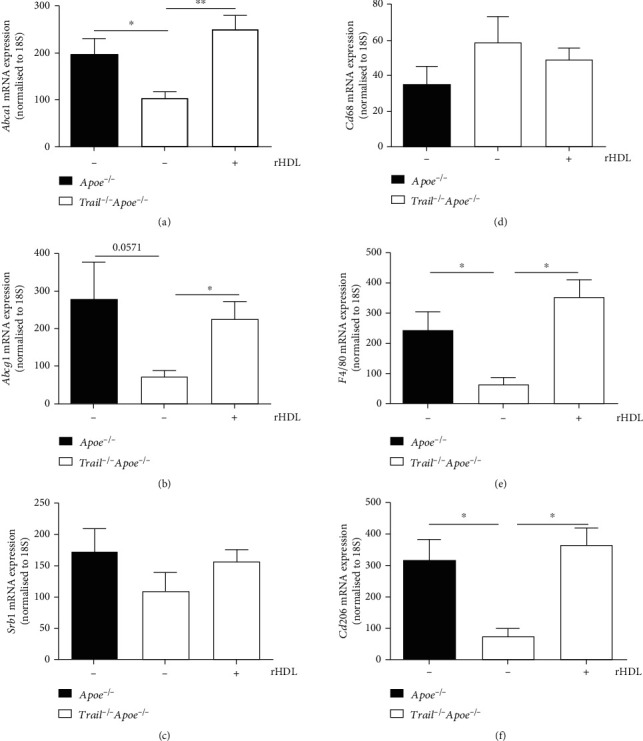
rHDL alters expression of genes regulating cholesterol metabolism and inflammation. *Trail^−/−^Apoe^−/−^* and *Apoe^−/−^* mice were fed a high-cholesterol diet for 12 weeks. In the last 2 weeks of the study, mice received 3 weekly infusions of either rHDL (20 mg/kg) or PBS. (a) *Abca1*, (b) *Abcg1*, (c) *Srb1*, (d) *Cd68*, (e) *F4/80*, and (f) *Cd206* mRNA expression in aortae. Aortae were isolated and mRNA extracted as described in Methods. mRNA expression was normalised to 18S (*n* = 4-6/group). Results are expressed as mean ± SEM; ^∗^*p* < 0.05 and ^∗∗^*p* < 0.01, ANOVA used in all conditions. rHDL: reconstituted high-density lipoprotein; Apoe: apolipoprotein E; TRAIL: TNF-related apoptosis-inducing ligand.

ABCA1, ABCG1, and scavenger receptor class B type 1 (SRB1) promote cholesterol efflux and exchange of cholesterol to influence atherosclerosis in mice [32–34]. The removal of excess cholesterol from macrophage foam cells by HDL is thought to be one of the key mechanisms underlying the atheroprotective properties of HDL [35], mediated primarily by ABCA1 and ABCG1 [36, 37]. There were no obvious differences in mRNA expression of hepatic *Abca1*, *Abcg1*, and *Srb1* in 12 w Western diet-fed *Trail^−/−^Apoe^−/−^* and *Apoe^−/−^* mice (Figure 3); however, aortic expression of *Abca1* and *Abcg1* was significantly decreased, >2-fold with TRAIL deletion, whereas *Srb1* mRNA levels remained unchanged (Figures 4(a)–4(c)). In contrast, rHDL infusions restored the reduced *Abca1* and *Abcg1* mRNA levels to those of control *Apoe^−/−^* mice (Figures 4(a) and 4(b)). These suggest that rHDL improves cholesterol homeostasis in *Trail^−/−^Apoe^−/−^* by modulating the expression of ABCA1 and ABCG1.

CD68 and F480 are macrophage markers. Because *Trail^−/−^Apoe^−/−^* mice have increased inflammation and macrophage accumulation in atherosclerotic lesions [25, 38], we examined changes to these markers from vascular tissues between *Trail^−/−^Apoe^−/−^* and control mice. An increased but not significant change in *Cd68* mRNA expression was observed in *Trail^−/−^Apoe^−/−^* aortae (Figure 4(d)). In contrast, a marked reduction, ~70%, in aortic *F480* mRNA levels was found in mice lacking TRAIL when compared to *Apoe^−/−^* (Figure 4(e)). Strikingly, *Cd206* mRNA levels mimicked those of *F480*, with *Trail^−/−^Apoe^−/−^* aortae expressing ~70% less compared with *Apoe^−/−^* aortae (Figure 4(f)). CD206 is expressed on alternatively activated or M2 macrophages [39]. Remarkably, rHDL infusions restored both *F480* and *Cd206* mRNA expression in *Trail^−/−^Apoe^−/−^* aortae to levels observed in control mice (Figures 4(e) and 4(f)). These findings suggest that rHDL may modulate inflammation and macrophage phenotype in atherosclerotic *Trail^−/−^Apoe^−/−^* mice.

## 4. Discussion

Although epidemiological studies have shown that low HDL levels correlate with increased risk of diabetes mellitus and cardiovascular disease [40, 41], preclinical and clinical studies aimed at raising HDL levels as a potential therapeutic to reduce diabetes and cardiovascular complications have been conflicting [42]. It is now known that HDL has pleiotropic functions, and HDL preparations can have multiple different effects on disease. Here, we sought to identify whether rHDL could improve features of diabetes-associated atherosclerosis using Western diet-fed *Trail^−/−^Apoe^−/−^* mice. We identified 3 novel findings. First, rHDL reduced plasma glucose and insulin levels and improved glucose and insulin tolerance in these mice. Second, rHDL infusion reduced cholesterol and triglyceride levels observed, as well as restoring ABCA1 and ABCG1 mRNA in the vessel wall. Third, atherosclerosis was only reduced in *Trail^−/−^Apoe^−/−^* mice with diabetic features, but not *Apoe^−/−^* mice. Collectively, these suggest that rHDL therapy could benefit patients with diabetes-associated atherosclerosis, and a more targeted approach may be necessary for treatment.

The beneficial effects of HDL on diabetes and changes to metabolic disease are well known. For example, mice deficient in ApoA-I—the main protein component on HDL—have impaired glucose tolerance compared to wild-type mice [43]. Furthermore, rHDL inhibited *β*-cell apoptosis and enhanced insulin secretion *in vitro*^16, 17^, and ApoA-I treatment of insulin-resistant mice increased insulin secretion and glucose clearance [44]. Indeed, intravenous infusions of rHDL to patients with type 2 diabetes stimulated glucose uptake into skeletal muscle, reduced plasma glucose levels, and increased insulin secretion [45] confirming preclinical findings. rHDL treatment in *Trail^−/−^Apoe^−/−^* mice also supports this view. We showed that improved glucose tolerance and insulin sensitivity occur in part, due to rHDL's anti-inflammatory effects on pancreata; rHDL reduced macrophage accumulation and increased insulin expression in *β*-cells in islets of Western diet-fed *Trail^−/−^Apoe^−/−^* mice.

In addition to type 2 diabetic features, *Trail^−/−^Apoe^−/−^* mice have significantly increased total cholesterol and triglycerides as well as increased VLDL and LDL compared to Western diet-fed *Apoe^−/−^* mice [25]. HDL's primary role is to mediate cholesterol efflux from atherosclerotic plaques, initially through interactions with ABCA1, then after remodelling through cholesterol exchange via SR-BI and ABCG1 [46]. Indeed, a deficiency or inhibition of *Abca1* and *Abcg1* enhanced cholesterol accumulation in atherosclerotic plaque via enhanced inflammation and monocyte/macrophage infiltration [47, 48]. Furthermore, *Trail*-negative macrophages had reduced cholesterol efflux, in part due to reduced ABCA1 and ABCG1 mRNA expression when compared with *Trail*-positive macrophages [38]. In the current study, ABCA1 and ABCG1 mRNA expression was significantly reduced in the vascular tissues of *Trail^−/−^Apoe^−/−^* mice, and importantly, rHDL administration restored this expression back to control levels. Because plaque from *Trail^−/−^Apoe^−/−^* mice have increased monocyte/macrophage accumulation and inflammation and considering that ABCA1 and ABCG1 mRNA was unaltered in the liver of *Trail^−/−^Apoe^−/−^* mice with rHDL infusion, rHDL may target lipid-laden macrophages in the vessel wall. This may also be relevant to lipid-laden macrophages in pancreata since downregulation of ABCA1 was associated with cholesterol accumulation in pancreatic *β*-cells, greater inflammation, and reduced insulin secretion [41]. However, this requires further elucidation.

A large number of preclinical studies of atherosclerosis suggest that HDL intervention reduces plaque size and inflammation [11, 49, 50]. We also observed that rHDL-treated *Trail^−/−^Apoe*^*-/*-^ mice had significantly reduced atherosclerotic plaque size compared to mice that received PBS; however, no changes were observed in atherosclerotic *Apoe^−/−^* mice. This is in contrast with early studies where infusing rHDL in cholesterol-fed rabbit models attenuated development of atherosclerosis [8, 9] and in *Apoe*-deficient mice where single infusions of HDL containing ApoA-I (apolipoprotein A-I)_Milano_ and dipalmitoyl phosphatidylcholine reduced plaque size, lipid, and macrophage content in atherosclerotic plaques [50]. As a caveat to these findings, other animal and HDL-targeted gene therapy studies demonstrated that increasing HDL with either infusions or overexpression of ApoA-I did not reduce the formation of atherosclerotic lesions but rather remodelled more advanced preexisting lesions similar to what is observed in human plaques as a more stable plaque phenotype [11, 51, 52]. Diabetic patients with obstructive coronary disease are known to have multiple metabolic features including elevated levels of metabolic stress markers [53], endothelial dysfunction [54], and increased inflammation in pericoronary adipose tissue [55], highlighting the complexity of the disease and its resulting cardiovascular complications. High fat diet-fed mice lacking TRAIL have many of these contributing metabolic changes including adiposity [25], endothelial dysfunction [56], and alterations in metabolic function [25, 57]. These are known to associate with atherosclerosis and CVD. Our findings highlight that rHDL treatment may be more useful in CVD patients with metabolic dysfunctions.

In summary, we have highlighted the importance of using a physiologically relevant diabetes-associated atherosclerosis model to investigate therapeutic strategies. In this model, HDL reduced features of diabetes associated with atherosclerosis. With much controversy surrounding the use of HDL in cardiovascular disease and the contradicting studies in preclinical models, this study identifies a continuing need for HDL research and a possible role for its use in targeted therapeutic strategies in people with diabetes-accelerated atherosclerosis.

## Figures and Tables

**Table 1:** Primer sequences.

Primer name	Forward 5′–3′	Reverse 5′–3′
ABCA1	AAAACCGCAGACATCCTTCAG	CATACCGAAACTCGTTCACCC
ABCG1	CGAGAGGGCATGTGTGACG	CCGAGAAGCTATGGCAACC
SR-B1	TTTGGAGTGGTAGTAAAAAGGGC	TGACATCAGGGACTCAGAGTAG
LDLR	TGACTCAGACGAACAAGGCTG	ATCTAGGCAATCTCGGTCTCC
TRAIL	CAGGCTGTGTCTGTGGCTGT	TGAGAAGCAAGCTAGTCCAATTTTG
SREBP-1	AGCAGCCCCTAGAACAAACAC	CAGCAGTGAGTCTGCCTTGAT
*β*-Actin	AACCGTGAAAAGATGACCCAGAT	CACAGCCTGGATGGCTACGTA
CD68	CCATCCTTCACGATGACACCT	GGCAGGGTTATGAGTGACAGTT
F4/80	CTTTGGCTATGGGCTTCCAGTC	GCAAGGAGGACAGAGTTTATCGTG
CD206	CAGGTGTGGGCTCAGGTAGT	TGTGGTGAGCTGAAAGGTGA

## Data Availability

Data are available on request.

## Supplementary Materials

Supplementary Materials Supplementary Figure 1: Western diet-fed *Trail^−/−^Apoe^−/−^* mice have impaired glucose tolerance compared to *Apoe^−/−^* mice. Supplementary Figure 2: HDL has no effect on glucose and insulin tolerance in *Apoe^−/−^* mice. Supplementary Figure 3: rHDL has no effect on plasma cholesterol, plasma triacylglycerol, or atherosclerosis in 12 w Western diet-fed *Apoe^−/−^* mice.
