# Supplementary material for: HDL Improves Cholesterol and Glucose Homeostasis and Reduces Atherosclerosis in Diabetes-Associated Atherosclerosis
Source: J Diabetes Res. 2021 May 6;2021:6668506. doi: 10.1155/2021/6668506 (PMC8163542; doi:10.1155/2021/6668506)
Supplement: Supplementary Materials — Supplementary Figure 1: Western diet-fed Trail−/−Apoe−/− mice have impaired glucose tolerance compared to Apoe−/− mice. Supplementary Figure 2: HDL has no effect on glucose and insulin tolerance in Apoe−/− mice. Supplementary Figure 3: rHDL has no effect on plasma cholesterol, plasma triacylglycerol, or atherosclerosis in 12 w Western diet-fed Apoe−/− mice. [file 6668506.f1.docx]

**Supplementary Material**

**HDL improves cholesterol and glucose homeostasis and reduces atherosclerosis in diabetes-associated atherosclerosis.**

Belinda A. Di Bartolo^1,2,3,4^, Siân P. Cartland^2,3,4^, Scott Genner^3^, Pradeep Manuneedhi Cholan^3^, Melissa Vellozzi^3^, Kerry-Anne Rye,^4^ and Mary M. Kavurma^2,3,4^.

The University of Sydney, ^1^Kolling Institute and ^2^Faculty of Medicine and Health, Sydney, Australia

^3^Heart Research Institute, Sydney, Australia

^4^The University of New South Wales, Faculty of Medicine, Sydney, Australia

***Supplementary Figure 1. Western diet-fed Trail^-/-^Apoe^-/-^ mice have impaired glucose tolerance compared to Apoe^-/-^ mice.***

*Trail^-/-^Apoe^-/-^* and *Apoe^-/-^* mice were fed a high cholesterol diet for 12 weeks. In the last 2 weeks of the study mice received 3 weekly infusions of either rHDL (20 mg/kg) or PBS. At euthanasia (A) fasting plasma glucose levels in 12 w Western diet-fed *Trail^-/-^Apoe^-/-^* vs. *Apoe^-/-^* mice (n= 8-10) were assessed. (B) A glucose tolerance test was performed; *Trail^-/-^Apoe^-/-^* (squares) and *Apoe^-/-^* (circles) at week 12 (n=6-7). Results are expressed as mean±SEM; **p*<0.05, ***p*<0.01 and *****p*<0.0001, Mann-Whitney *t* test and Two-way ANOVA with Bonferroni correction. Apoe; Apoliprotein E; TRAIL; TNF-related apoptosis-inducing ligand.

A

B

***Supplementary Figure 2. HDL has no effect on glucose and insulin tolerance in Apoe^-/-^ mice***

*Apoe^-/-^* mice were fed a high cholesterol diet for 12 weeks. In the last 2 weeks of the study mice received 3 weekly infusions of either rHDL (20 mg/kg) or PBS. (A) glucose tolerance test and (B) insulin tolerance test were performed as described in the methods; PBS-treated *Apoe^-/-^* (circles) and rHDL-treated *Apoe^-/-^* (squares) at week 12 (n=6-8). Results are expressed as mean±SEM. Apoe; Apoliprotein E, rHDL; reconstituted high-density lipoprotein.

A

B

***Supplementary Figure 3. rHDL has no effect on plasma cholesterol, plasma triacylglycerol or atherosclerosis in 12 w Western diet-fed Apoe^-/-^ mice.***

*Apoe^-/-^* mice were fed a high cholesterol diet for 12 weeks. In the last 2 weeks of the study mice received 3 weekly infusions of either rHDL (20 mg/kg) or PBS. (A) Fasting plasma cholesterol (n=7-8) and (B) triacylglycerol levels (n=7-8) at euthanasia from Western diet-fed *Apoe^-/-^* mice. (C) Plaque area/total artery area is unaffected with rHDL infusion (n= 4-6). Results are expressed as mean±SEM. Apoe; Apoliprotein E, rHDL; reconstituted high-density lipoprotein.

**A**

**B**

**C**
